# Environmental regulation and financial performance of Chinese listed companies

**DOI:** 10.1371/journal.pone.0244083

**Published:** 2020-12-28

**Authors:** Bing Zhou, Jing Wu, Sidai Guo, Mingxia Hu, Jing Wang

**Affiliations:** 1 Research Center for Economy of Upper Reaches of the Yangtse River/School of Accounting, Chongqing Technology and Business University, Chongqing, China; 2 Sichuan Province Circular Economy Research Center, Southwest University of Science and Technology, Sichuan, China; 3 Accounting Research and Development Center, Beijing National Accounting Institute, Beijing, China; 4 School of Law, Lanzhou University of Technology, Lanzhou, China; Institute for Advanced Sustainability Studies, GERMANY

## Abstract

**Objective:**

The answer to this article lies in: Does the financial activities of physical enterprises have an adverse impact on their main business? Is it conducive to the sustainable development of the national economy? However, when most scholars study the impact of environmental regulations on companies performance, they have not classified companies performance. This article will study the relationship between environmental regulations and performance levels based on the classification of companies performance, and then divide the nature of industry pollution, companies location and nature of property for in-depth research.

**Methods:**

First, this article uses a random effect variable-intercept model to measure companies financial performance and non-financial performance. Then, the variables are divided into two variable groups: light pollution and heavy pollution according to the nature of industry pollution. Next, the companies are divided into three variable groups: the eastern region, the central region, and the western region. Finally, the company is divided into two variable groups: state-owned and non-state-owned according to the nature of property.

**Conclusions:**

The study found that: (1) Environmental regulations have inhibited companies financial activities. And the inhibitory effect of environmental regulations on the financial performance of enterprises is more obvious in the heavily polluting industries and enterprises in central and eastern regions. (2) Environmental regulations and companies non-financial performance are also negatively related, environmental regulations have also inhibited the non-financial performance of companies, this effect is more pronounced in heavily polluting industries and enterprises in western regions. (3) Income crowding effect brought by China's environmental regulations is greater than the income compensation effect brought by stimulating technological innovation.

## Introduction

While rapid reform and development have brought abundant material life, at the same time, it has also been accompanied by population, resource, and environmental issues. China promulgated and implemented the first environmental law "Environmental Protection Law (Trial)" in 1979. Up to now, environmental laws have a constitution, 14 laws, and 26 administrative regulations [1]. The promulgation of these laws and regulations truly embodies the substantive transformation of China's environmental rule of law from nothing to comprehensive, and witnesses the change in people's awareness of environmental concepts in the process of China's economic development. At the 13th National People’s Congress, ‘Construction of ecological civilization’ was written into the ‘Constitution’. The state power organs are also committed to regulating social and companies behaviors from top to bottom through a perfect system. All actions have revealed the country's firm determination and confidence in exploring the sustainable development relationship between the ecological environment and economic balance.

In recent years, similar to environmental issues, companies financial activities have also received widespread attention. As an important pillar of the real economy, the primary task of the manufacturing industry is to provide society with high-quality products. However, as the profit rate of Chinese entity industry has decreased, in recent years, a large number of listed domestic manufacturing companies have begun to actively invest in financial assets, such as purchasing bank wealth management products, restructuring asset, and buying and selling stocks in the secondary market. Manufacturing industry as the foundation of the country’s economic development and stability, the financial development trend of entity companies deserve great attention from the practitioners and academia. Does the financial activities of the entity companies has a negative impact on the main business? Is it conducive to the sustainable development of the national economy? These have become questions that the theoretical and practical circles must answer. This article takes the financial data of China’s listed companies from 2014 to 2018 as the research object, and considers the background of increasing emphasis on environmental issues. In order to answer the above questions, it analyze the impact of environmental regulations on companies performance from the dual perspectives of the composition of companies performance (financial performance and non-financial performance).

The contribution of this article are: firstly, this article divides the performance of companies into financial performance and non-financial performance, and analyze the impact of environmental regulations on the performance of companies with different degrees of financial activities. Secondly, this article group companies based on the firms’ industry classification codes and then divide the companies according to the nature of industry pollution, companies location and nature of property for an in-depth analysis. It studies the regional, industry and property differences in the different degree of companies financial activities under the influence of environmental regulations.

## Environmental regulation and companies performance

### Three hypotheses

The research conclusions of domestic and foreign scholars on the relationship between environmental regulation and companies performance have mainly formed three views: traditional hypothesis, Porter hypothesis, and uncertainty hypothesis.

1. The traditional hypothesis believes that the increase in the intensity of environmental regulations will make companies have to allocate a portion of funds for environmental protection investments, thereby encroaching on companies funds. Rassier and Earnhart [2] found that the promulgation of strict emission environmental regulations will cause listed companies in the chemical manufacturing industry to reduce companies financial performance. Richard Kneller and Edward Manderson [3] conducted a study on the British manufacturing industry and found that the additional cost of environmental regulation by companies will have a crowding out effect on non-environmental expenditure and physical asset investment. Li Bin [4] studying the impact of environmental regulations on the contribution rate of economic growth, believing that environmental regulations reduce the productivity of enterprises and inhibit the contribution rate of economic growth. Yu Wei, Chen Qiang, and Chen Hua [5] studied 37 industrial industry data from 2003 to 2010 and found that although environmental regulations can induce technological innovation in industrial enterprises, but the effects of technological innovation are not enough to make up for the promotion of environmental input on the performance of industrial enterprises.
2. The Porter hypothesis puts forward a different point of view. Strict environmental policies will force companies reforms, promote companies efficiency and encourage innovation, thereby enhancing companies competitiveness and companies performance. Zhao Hong [6] studied the data of 18 industries in China from 1996 to 2004 and found that environmental regulations had a significant positive effect on R&D expenditures and the number of patent applications lagging three phases. The Porter hypothesis was partially confirmed. Eiadat [7] survey data on the chemical industry in Jordan shows that environmental innovation strategies can improve company business performance. Yang Shuting, Zeng Gang [8] study the ecological innovation effect of environmental regulation from the perspective of regional differences, the study found that there is a "U"-shaped relationship between regulatory intensity and ecological innovation. Factors such as environmental regulation, economic development level, R&D investment and other factors have threshold effects. Only when these thresholds are crossed, the Porter hypothesis can be established.
3. The uncertainty hypothesis holds that there is an insignificant relationship between environmental regulations and companies performance. Shen Neng and Liu Fengchao [9] used the non-linear threshold panel model from the national and regional levels to find that there are regional differences in the promotion of technological innovation by environmental regulations. The Porter hypothesis is difficult to support in central and western China. Jiang Ke [10] uses panel data from 20 pollution-intensive companies in China to study that environmental regulations have a significant impact on companies in heavy-polluting industries, but they have no significant effect on companies in medium- and light polluting industries. Peng Cong and Yuan Peng [11] used the environmental regulation intensity index of each province from 2007 to 2014 and found that economic growth and environmental regulation intensity present a non-linear "inverted U" relationship, and environmental regulation has a significant positive spatial spillover effect.

### Two perspectives

In addition to the above three hypotheses, scholars at home and abroad usually have two perspectives on the relationship between environmental regulation and companies performance, one is the effect of pollution avoidance, and the other is the effect of innovation compensation.

1. Pollution avoidance effect mainly refers to the tendency of enterprises in pollution-intensive industries to establish plant sites in countries or regions with relatively low environmental standards. Fu Jingyan and Li Lisha [12] constructed industrial environmental regulation indicators and industrial pollution density indicators. They studied the data of 24 Chinese manufacturing industries from 1996 to 2004 an d concluded that my country's pollution-intensive industries do not have absolute advantages. Environmental regulation indicators have a negative impact on comparative advantage. The quadratic term of environmental regulation has a positive influence on comparative advantage. Environmental regulation and comparative advantage present a "U"-shaped relationship. Ji Hong and Liu Ying [13] found that when environmental regulations are made as endogenous variables, the empirical results show that the significance of the pollution avoidance effect test will be greatly enhanced, and the entry of Chinese foreign investment is positively correlated with the level of environmental regulations. Zheng Yue [14] used city-level data and used a Poisson model to investigate the impact of environmental regulations on the site selection of new Chinese manufacturing companies. The study found that the “pollution avoidance” effect is very significant, and environmental regulations have a significant impact on the entry of new companies. Yang Xin [15] analyzed the spatial distribution pattern of polluting companies in China and used spatial measurement methods to examine the location of polluting companies on national, provincial, and prefecture-level city boundaries, and found that there are obvious boundary effects. Xu Zhiwei and Liu Chenshi [16] used the specific geographic information of companies in the Beijing-Tianjin-Hebei region from 2005 to 2012 to find out that polluting companies will eventually gather around cities with low environmental regulatory intensity standards and set up factories, forming a pollution “gray edge” surrounding the central city.
2. The innovation compensation effect is that environmental regulations lead to enterprise innovation. The theory holds that the improvement of economic performance can compensate the environmental cost to a certain extent. The research of Jaffe, Adam B., Palmer, Karen [17] found that the lagging environmental regulation expenditure of enterprises promotes the R&D expenditure of enterprises. Ar I M, Baki B. [18] used the data provided by 270 managers of small and medium-sized enterprises in Turkish science and technology parks and found that green product innovation has a clear positive relationship with business performance. Feng Yuxia [19] explained the relationship between China's regional environmental regulations and total factor productivity from 1992 to 2008. The research found that China's total factor productivity was rising, mainly relying on technological progress. Li Weihong, Bai Yang [20] believe that in a competitive environment, companies with high R&D efficiency, knowledge absorption level and technological achievement conversion rate can obtain economic performance through technological innovation under the incentive of government subsidy policies to compensate for environmental management costs. Yao Xiaojian, He Shan, and Yang Guanglei [21] empirically verify that environmental regulations and technological progress have a significant "U"-shaped relationship.

### A brief comment

After reviewing the aforementioned literature, it is found that the research on the impact of environmental regulations on companies performance has three main conclusions: First, environmental regulations are not conducive to the improvement of companies performance. Second, environmental regulations promote companies performance; third, the impact of environmental regulations on companies performance is uncertain. From these literature, it is also found that these scholars did not classify the performance of enterprises when studying the impact of environmental regulations on companies performance. Therefore, on the basis of classifying companies performance, this article divide the companies according to the nature of industry pollution, companies location and nature of property for in-depth research.

## Theoretical analysis and hypothesis

### Intensity of environmental regulations and companies financial performance

Government and enterprises are the two major sectors of the economy. In terms of environmental regulations, the government can formulate companies emission standards and collect pollution taxes from a macro level. Enterprises are the cornerstone of the country's economic development. If the pollution discharge standard is high, it may affect the performance of the enterprise. According to the division of financial activities and non-financial activities, companies performance can be divided into financial performance and non-financial performance. Regarding the relationship between environmental regulations and companies financial performance, China is currently in a period of industrial transformation. Under the dual pressure of market and environmental regulations, there are obstacles to companies financing and investment. From a market perspective, investors and banks have more sensitive risk awareness, and it is more difficult for companies with poor market development to raise funds; From the perspective of environmental regulations, the government implements development restrictive measures for companies with serious environmental pollution problems, which will also lead to limited investment channels and reduced investment opportunities.

From the perspective of companies pollution, for heavy polluting enterprises, strict environmental regulations will lead to severe restrictions on their investment decisions. In comparison, light polluting enterprises have relatively quick response capabilities under the pressure of environmental regulations.

From the perspective of companies location, compared with the central and eastern regions, the development potential of the western region has not been fully released and the potential labor supply is sufficient. In addition, in recent years, the state has given many preferential policies that special support to the western region. The gradual development of the western region has created good conditions for companies financing and investment, which is conducive to companies in the western region to obtain financial investment income in financial activities.

From the perspective of companies property, environmental regulations may have different effects on the companies performance of different property rights. Compared with state-owned enterprises, due to the higher relative value of environmental protection investment scale of non-state-owned enterprises, the financial performance of non-state-owned enterprises is more sensitive to the response of environmental regulations.

Based on the above analysis, the following hypotheses are proposed:

H1: Under control of other conditions, environmental regulations will inhibit companies financial performance.
H1a: Compared with light polluting enterprises, environmental regulations have a more significant inhibitory effect on heavy polluting enterprises.
H1b: Compared with the western region, environmental regulations have a more significant inhibitory effect on the central and eastern enterprises
H1c: Compared with state-owned enterprises, environmental regulations have a more obvious impact on the financial performance of non-state-owned enterprises

### Intensity of environmental regulations and companies non-financial performance

Companies non-financial performance mainly refers to the business performance of entity companies. In recent years, due to the high standards of environmental protection, most companies face high demand for environmental protection investment such as the construction of environmental protection facilities and the development of resource utilization technologies. Environmental protection investment is expensive and has a long cycle time, which will squeeze the company investment in other new production economic projects, thereby affecting company non-financial performance. At the same time, the extensive economic development model that relies on resources for a long time has led to insufficient investment in technological innovation of enterprises. Existing equipment and technology are difficult to support the resource-intensive development model, which affects the improvement of long-term performance. Therefore, strict environmental regulations will speed up the internalization of external costs and affect companies’ performance. Strict environmental regulation measures will put greater economic pressure on the transformation of heavily polluting enterprises, and high environmental protection investment will affect the production and operation of companies and even be forced to withdraw from the market.

From the perspective of companies pollution, for light polluting enterprises, environmental regulations can promote green production activities, further expand product markets, enhance customer satisfaction, and bring compensation effects through companies innovation.

From the perspective of companies location, different regions have different impacts of environmental regulations on the non-financial performance of enterprises. Due to the high level of economic development, strong capital accumulation, more advantageous location conditions, and more talent gathering in the eastern region of China, there are more ways to respond when facing environmental regulatory pressures, that will help companies to offset the inhibitory effects of environmental regulations.

From the perspective of companies property, the impact of environmental regulations on the non-financial performance of companies with different property rights may be different. However, in the medium and long term, environmental regulations may inhibit the main performance of state-owned and non-state-owned enterprises.

Based on the above analysis, the following hypotheses are proposed:

H2: Under control of other conditions, environmental regulations will inhibit the non-financial performance of enterprises.
H2a: Compared with light polluting enterprises, environmental regulations have more significant restraint effects on heavy polluting enterprises.
H2b: Compared with the central and eastern regions, environmental regulations have a more significant inhibitory effect on western enterprises.
H2c: Environmental regulations have a restraining effect on the non-financial performance improvement of state-owned and non-state-owned enterprises.

## Methodology and data

### Data selection

The data required in this article comes from the CSMAR database of China's A-share listed companies in the five years from 2014 to 2018. In order to ensure the validity of the data, follow the steps below to screen the original samples:

1. Exclude listed companies with ST or *ST during the period
2. Eliminate listed companies with incomplete financial data
3. Eliminate missing samples
4. Exclude the sample of listed financial companies

After screening, 14196 sample values of 2,839 listed companies were finally obtained. In order to eliminate the influence of extreme values, this paper narrows down all continuous variables at the 1% and 99% points.

The industry classification refers to the 2012 industry classification guidelines of the China Securities Regulatory Commission and is classified according to the first-level industry classification. All samples in this article are divided into 18 industries. Select 9 industries including manufacturing, electricity production industry, mining industry, real estate, comprehensive, construction industry, transportation, storage and post industry, accommodation industry, primary industry as a heavily polluting enterprise. Select 9 industries including environment management industry, wholesale and retail industry, health and social work, entertainment industry, information technology service industry, education, scientific service industry, leasing and business services industries, other service industries as light pollution industries. (There is ‘no other service industry’ in the enterprise data contained in this article.)

At the same time, according to China's regional division standards, the company is divided into three regions: East, Central and West. Among them, the eastern region includes 11 provinces and cities: Beijing, Tianjin, Shandong, Guangdong, Hainan, Hebei, Liaoning, Shanghai, Jiangsu, Zhejiang, Fujian. The central region includes 10 provinces and cities: Shanxi, Jilin, Henan, Hubei, Hunan, Heilongjiang, Anhui, Jiangxi, Guangxi, Inner Mongolia. The western region includes 10 provinces and cities: Xinjiang, Ningxia, Qinghai, Gansu, Shaanxi, Tibet, Yunnan, Guizhou, Chongqing, Sichuan.

### Variable selection

#### Financial performance (RFI)

The financial activities of entity enterprises mainly include: direct or indirect purchase of stocks, bonds and other securities; purchase indirect financing securities issued by financial intermediaries such as banks, trusts or securities firms; implement inter-enterprise entrusted loans, etc.

In this paper, the financial performance is measured based on the investment income in the income statement. Here, the financial performance measurement index is constructed as:

RFI=ln(Investmentincome-Incomeobtainedorconfirmedfromjointventuresorassociates+Changesinfairvalue)

#### Non-financial performance (RO)

The non-financial performance of a company is mainly derived from the income of its main business, which is reflected in the operating profit index in the companies income statement. Here, the non-financial performance measurement index is constructed as:

RO=ln(Operatingincome-Financialperformance)

#### Environmental Regulations (ER)

According to the analysis of the existing literature, environmental regulations are divided into explicit environmental regulations and implicit environmental regulations. Among them, explicit environmental regulations refer to binding regulations that exist in tangible forms, such as laws, agreements and regulations, with environmental protection as the goal, individuals and organizations as the targets of regulation. In practice, the commonly used policy measures for environmental regulation can be divided into three categories: command-control measures, economic measures and encouragement measures. Command-control measures are the government's use of coercive force to control the behavior of enterprises that harm the environment, including stipulating production technology, setting emission quotas, and setting pollutant emission standards, etc. It has the advantages of accurate problem location, easy operation and quick results, and is widely used by environmental management departments in various countries. Economic measures are to adjust the economic interest relationships of all parties in the market through economic levers, and to encourage enterprises to protect the environment. Encouragement measures are not characterized by law enforcement, but through public environmental education, providing companies with environmental information, guiding companies to sign voluntary agreements, etc., and work together to achieve the goal of improving the environment.

This article mainly focuses on the government's command-control environmental regulations. The main measurement indicators are: companies pollution control costs, three-simultaneous environmental protection investment, and the number of environmental administrative punishment cases accepted during the year. In order to facilitate the quantification of indicators, environmental regulation mainly selects the cost of companies pollution control costs for measurement.

Refer to the research of Lou Changlong and Ran Maosheng [22] to construct environmental regulation indicators:

$$ER=\frac{Investment\;in\;regional\;pollution\;control}{Regional\;GDP}*1000$$

#### Control variables

To ensure the stability and effectiveness of the model constructed in this article, the following control variables are selected in this article:

1. Nature of property (State)
2. Enterprise size (Size)
3. Enterprise growth (Growth)
4. Return on equity (Roe)
5. Major shareholders' capital possession (Occupy)
6. Leverage ratio (Lev)

See Table 1 for specific definitions of the above variables.

**Table 1:** Summary of variables.

The variable type, variable name, symbol and definition.			
Variable type	Variable name	Symbol	Definition
Explained variable	Financial performance	RFI	The natural logarithm of the value of investment income minus the investment income of associates or joint ventures and the gains and losses from changes in fair value.
	Non-financial performance	RO	The natural logarithm of operating income minus financial performance value
Explanatory variables	Environmental regulation	ER	Regional pollution control investment divided by regional GDP
Control variable	Nature of Property	State	Assign a value of 1 to state-owned enterprises and 0 to non-state-owned enterprises
	Enterprise size	Size	The natural logarithm of the company's total assets
	Enterprise growth	Growth	Main business income growth rate (control analysis based on 25, 50, and 75 points)
	Return on equity	Roe	Net profit divided by shareholders' equity
	Major shareholders' capital possession	Occupy	Other receivables divided by total assets at the end of the year
	Leverage ratio	Lev	Total liabilities at the end of the period divided by total assets at the end of the period
	Industry variables	Ind	Guided by the division of industries in the national economy, subdivide 18 industries
	Annual variable	Year	Based on 2014, a total of five annual dummy variables

This article sets up annual dummy variables (Year) and industry dummy variables (Ind) to control the annual and industry effects.

### Model construction

In order to test Hypothesis 1 and Hypothesis 2, panel data of 2,839 listed companies from 2014 to 2018 are used for empirical analysis. Because the sample data has a large cross-section (N = 2839) and a small time series (T = 5), and the model contains variables that need to be explained that reflect the difference between individual financial performance and non-financial performance, so the variable intercept panel data is selected. Based on the above reasons, a random effect variable intercept model is established, as follows:

#### (1) Financial performance (RFI)

In order to test the hypotheses H1, H1a, H1b, H1c, construct the environmental regulation model 1 as follows:

$${\text{RFI}}_{\text{i},\text{t}}=\beta_{0}+\beta_{1}{\text{ER}}_{\text{i},\text{t}}+\sum {\text{KControl}}_{\text{i},\text{t}}+{\text{v}}_{\text{i}}+{\text{u}}_{\text{i},\text{t}}$$

In the model, β_0_ represents the average financial performance level; v_i_ is an unobservable factor that reflects the financial performance level of the entity company, and u_i,t_ is a random error term.

#### (2) Non-financial performance (RO)

In order to test the hypotheses H2, H2a, H2b, H3c, construct the environmental regulation model 2 as follows:

$${\text{RO}}_{\text{i},\text{t}}=\beta_{0}+\beta_{1}{\text{ER}}_{\text{i},\text{t}}+\sum {\text{KControl}}_{\text{i},\text{t}}+{\text{v}}_{\text{i}}+{\text{u}}_{\text{i},\text{t}}$$

Variable consistency is defined and is the same as model 1.

## Empirical results and discussion

### Descriptive statistics

Table 2 shows the descriptive statistical results of the main variables. The statistical indicators included are mean, standard deviation, minimum and maximum. According to descriptive statistics, the average value of companies’ financial performance (RFI) during the sample period is 12.37, the maximum value is 21.02, and the minimum value is 0. The distribution is not uniform. The average value of companies’ non-financial performance (RO) is 15.56, and the maximum value is 22.93, indicating that there is a large gap in non-financial performance of companies.

**Table 2:** Descriptive statistics.

Variable	Obs	Mean	Std. Dev.	Min	Max
ER	14196	8.884	5.821	2.387	32.348
RFI	14196	12.367	7.074	0	21.02
RO	14196	15.56	7.35	0	22.934
Size	14196	22.181	1.286	19.887	26.109
Growth	14196	.166	.357	-.527	2.05
Lev	14196	.412	.202	.058	.876
Roe	14196	.074	.109	-.45	.368

Other control variable indicators include: The average enterprise size (Size) is 22.18, and the maximum is 26.10. The small gap indicates that the overall scale of Chinese enterprises is at a normal level; The minimum and maximum values of company growth (Growth) are -0.527 and 2.050, respectively. The large gap indicates that there is a gap in the business growth of domestic companies; The minimum and maximum values of major shareholders' capital possession (Occupy) are relatively small, but the difference is large, indicating that the capital occupation by major shareholders of most companies in China is within the normal range, and and there are differences in the degree of capital occupation among companies; The average leverage ratio (Lev) is 0.401, indicating that the overall financial leverage of Chinese companies is at a normal level, and the large gap between the maximum and minimum values indicates that there is a large gap between the leverage ratios; The large gap between the maximum and minimum values of return on equity (Roe) indicates that there are a large gap between companies.

**Fig 1:**
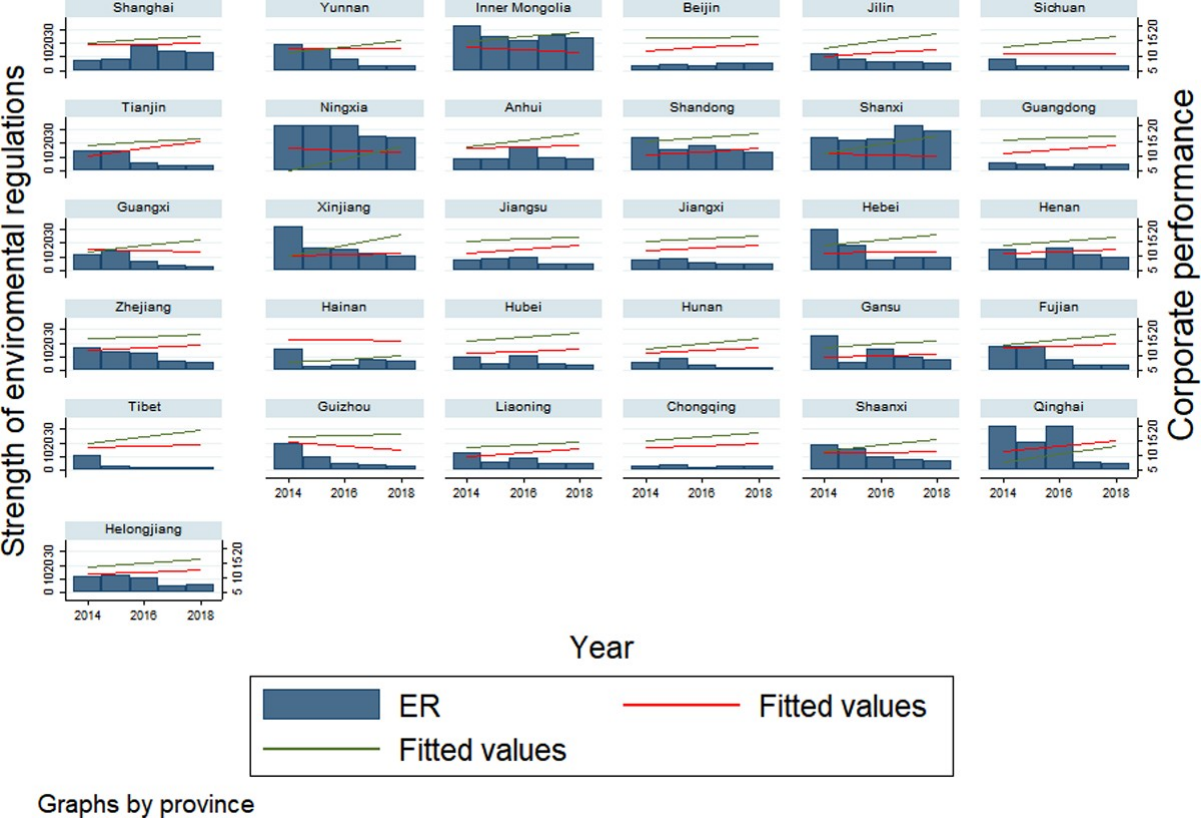
Environmental regulations and performance levels in provinces.

Fig 1 is a time series figure of the intensity of environmental regulation and performance level. The bar chart in the figure reflects the intensity of environmental regulation in different provinces during the five-year period from 2014 to 2018. The red fitted line represents the financial performance time series line, and the green line represents non-financial performance time series line.

As shown in the Fig 1, the six provinces with strong environmental regulations are Ningxia, Inner Mongolia, Shanxi, Qinghai, Shandong, and Xinjiang. And the performance level of enterprises in most provinces decreases with the increase in the intensity of environmental regulations, showing a negative correlation. Most Provinces with a downward trend in the intensity of environmental regulations have an upward trend in performance.

**Fig 2:**
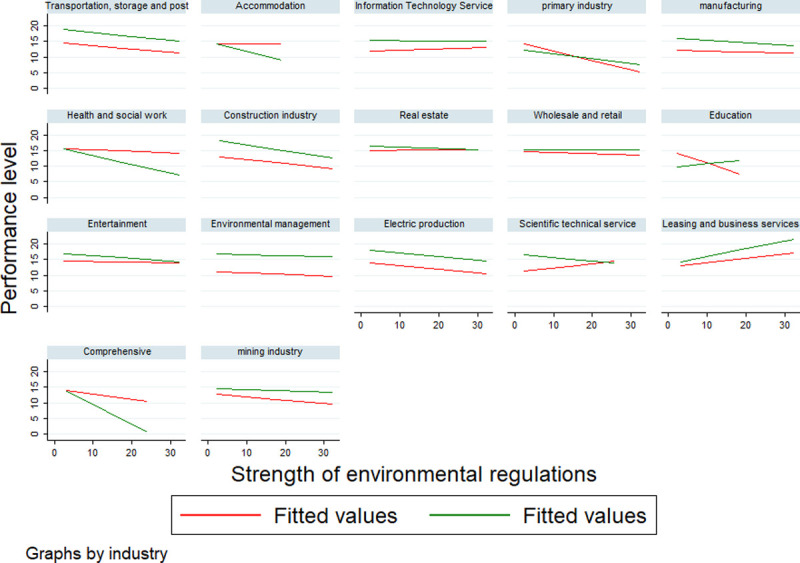
Environmental regulations and performance in the industry.

Fig 2 shows the relationship between environmental regulations and performance in each industry. The red fitted line represents the financial performance time series line, and the green line represents non-financial performance time series line. It shows that financial performance and non-financial performance of most industries are negatively correlated with the intensity of environmental regulations. However, the non-financial performance level of the education industry, the financial performance level of the scientific service industry, and the financial and non-financial performance level of the leasing and business service industries are obviously positive correlated.

**Fig 3:**
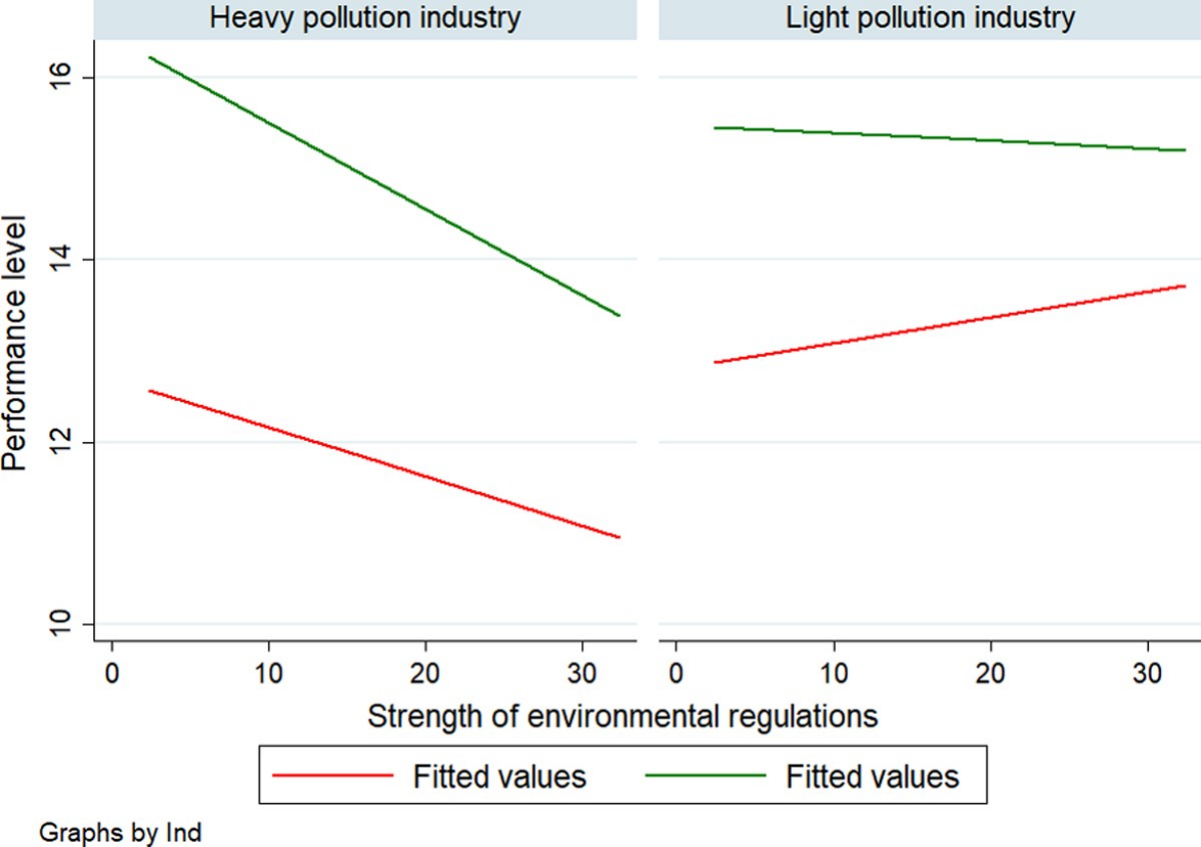
Environmental regulations and performance according to pollution degree.

Fig 3 is a diagram of the relationship between environmental regulations and performance levels after categorizing industries according to pollution degree and pollution nature. It can be seen that non-financial performance has significant advantages over financial performance. Compared with light polluting enterprises, the financial performance and non-financial performance of heavy polluting enterprises have a more obvious negative correlation with the intensity of environmental regulations. The non-financial performance of light polluting enterprises is slightly negative correlated with the intensity of environmental regulations, and the financial performance is positively correlated with the intensity of environmental regulations.

### Relevance statistics

Table 3 shows the correlation coefficients of the main variables. The correlation coefficient of environmental regulation (ER) to companies financial performance (RFI) is -0.037, indicating that the strength of environmental regulation inhibits financial performance, which has an inhibitory effect at the 1% confidence level; The correlation coefficient between environmental regulations and companies non-financial performance (RO) is -0.064, indicating that the strength of environmental regulations inhibits companies non-financial performance, which has an inhibitory effect at the 1% confidence level.

**Table 3:** Relevance statistics.

	RFI	RO	ER	Size	Growth	Lev	Occupy	Roe
RFI	1							
RO	-0.008	1						
ER	-0.037***	-0.064***	1					
Size	0.254***	0.168***	0.030***	1				
Growth	-0.024***	0.215***	-0.063***	0.018**	1			
Lev	0.00700	-0.122***	0.051***	0.549***	0.00700	1		
Occupy	0.060***	-0.156***	-0.026***	0.097***	-0.017**	0.228***	1	
Roe	0.080***	0.605***	-0.040***	0.069***	0.226***	-0.145***	-0.107***	1

Note: ***, **, * are significant at the level of 1%, 5% and 10% respectively.

For the control variables, most of the indicators of enterprise size, enterprise growth, leverage ratio, major shareholder’s capital possession, return on net assets are significant at the 1% level.

In addition, the correlation coefficient between variables is generally small, the maximum value is 0.605, and the maximum value of the variance inflation factor of the variables is 1.57, so there is no need to worry about multicollinearity.

### Regression analysis

#### Environmental regulations and companies financial performance

According to the previous research hypotheses, the model (1) is regressed to test the relationship between environment regulations and companies financial performance. The results are shown in Table 4.

**Table 4:** Environmental regulations and companies financial performance.

	(1)	(2)	(3)	(4)	(5)	(6)	(7)	(8)
RFI	Full sample	Heavy pollution industry	Light pollution industry	Eastern Region	Central Region	Western Region	State-owned	Non-state
ER	-0.044***	-0.049***	0.007	-0.047***	-0.089***	0.005	-0.021	-0.051***
	(-4.01)	(-4.07)	(0.28)	(-3.29)	(-2.99)	(0.24)	(-1.24)	(-3.61)
Size	1.997* **	1.933***	2.461***	2.095***	1.833***	1.715***	1.782***	2.186***
	(29.04)	(24.89)	(16.52)	(26.49)	(9.37)	(8.80)	(15.37)	(23.53)
Growth	-0.633***	-0.596***	-0.899***	-0.647***	-0.295	-0.972**	-1.183***	-0.488***
	(-4.20)	(-3.37)	(-3.18)	(-3.54)	(-0.79)	(-2.56)	(-4.30)	(-2.68)
Lev	-6.668***	-6.853***	-5.982***	-6.464***	-7.921***	-5.831***	-6.034***	-6.946***
	(-15.58)	(-13.80)	(-7.24)	(-12.80)	(-6.94)	(-5.14)	(-8.02)	(-13.21)
Occupy	19.826***	18.488***	19.618***	19.141***	12.522*	24.411***	18.322***	19.152***
	(7.11)	(5.63)	(3.72)	(5.64)	(1.77)	(3.58)	(4.02)	(5.41)
Roe	2.369***	2.496***	1.667	1.898***	0.458	5.802***	3.403***	1.909***
	(4.35)	(3.98)	(1.54)	(2.91)	(0.33)	(4.10)	(3.80)	(2.79)
_cons	-29.111***	-27.751***	-39.116***	-31.055***	-24.851***	-24.586***	-24.932***	-32.956***
	(-20.14)	(-17.07)	(-12.32)	(-18.72)	(-6.04)	(-5.92)	(-9.91)	(-16.95)
Ind	Control	Control	Control	Control	Control	Control	Control	Control
Year	Control	Control	Control	Control	Control	Control	Control	Control
N	14196	11443	2753	10059	2234	1903	4932	9264
R2	0.10	0.10	0.13	0.11	0.08	0.08	0.11	0.09

Note: ***, **, * are significant at the level of 1%, 5% and 10% respectively.

In Table 4, column (1) is the full-sample test result. The regression coefficient of environmental regulation intensity (ER) and companies financial performance (RFI) is -0.044, and it is significant at the 1% confidence level, which shows that government environmental regulation policies have a restraining effect on the financial performance of enterprises. The H1 hypothesis is verified.

Columns (2) and (3) are the regression results obtained after distinguishing the pollution degree and the pollution natures. The regression coefficient of environmental regulation (ER) and financial performance (RFI) of severely polluting industries is -0.049, which is significant at the 1% confidence level. The environmental regulation (ER) and financial performance (RFI) of light-pollution industries show an insignificant positive correlation, with a regression coefficient of 0.07. It can be seen that there are significant differences in the impact of government environmental regulations on enterprises with different pollution levels and pollution natures. The government's environmental regulatory pressure has brought significant performance suppression effects to heavy pollution industries, while it has an insignificant boost to the financial performance of light pollution industries. The H1a hypothesis is verified.

Columns (4), (5), and (6) are the results of sub-regional inspections. The regression coefficients of environmental regulations (ER) and financial performance (RFI) in the eastern, central, and western regions are- 0.047, -0.089, 0.005, respectively. Among them, the eastern region and the central region are both significant at the 1% confidence level, and the regression results of the western region are not significant. It can be seen that there are obvious regional differences in the effects of the government's environmental regulation policies, which have a significant inhibitory effect on the eastern and central regions. The reason is that the level of companies financial performance (RFI) is greatly affected by policy inclination. Compared with the central and eastern regions, the development potential of the western region has not been fully released. In addition, in recent years, many preferential policies have been given to the western region, which can’t be enjoyed in central and eastern provinces. The H1b hypothesis is verified.

Columns (7) and (8) are the regression test results of different property rights. The regression coefficients of environmental regulation (ER) and financial performance (RFI) for state-owned and non-state-owned are -0.021 and -0.051, respectively. Among them, the non-state-owned enterprises are significant at the 1% confidence level, and the state-owned regression results are not significant. It can be seen that there are obvious differences in different property rights in the effects of environmental regulatory policies, which have a significant inhibitory effect on non-state-owned enterprises. The H1c hypothesis is verified.

From the perspective of control variables, the enterprise size (Size), the major shareholder's capital occupation (Occupy), and the return on net assets (Roe) all have a positive correlation with the financial performance (RFI) of the enterprise at the 1% confidence level. Leverage ratio (Lev), companies growth (Growth) with companies financial performance (RFI) all show a negative correlation at the 1% confidence level. It shows that the larger the company, the higher the degree of the major shareholder's capital occupation, the higher the company's net asset income, and the easier it is to obtain good financial performance. However, it is not easy for companies with high leverage ratios or growth indicators to achieve good financial performance.

#### Environmental regulations and companies non-financial performance

According to the previous research hypotheses, the model (2) is regressed to test the relationship between environmental regulations and companies non-financial performance. The results are shown in Table 5.

**Table 5:** Environmental regulations and companies non-financial performance.

	(1)	(2)	(3)	(4)	(5)	(6)	(7)	(8)
RO	Full sample	Heavy pollution industry	Light pollution industry	Eastern Region	Central Region	Western Region	State-owned	Non-state
ER	-0.058***	-0.063***	-0.029	-0.044***	-0.053**	-0.079***	-0.072***	-0.041***
	(-6.49)	(-6.48)	(-1.28)	(-3.78)	(-2.26)	(-4.32)	(-4.59)	(-3.84)
Size	1.264***	1.240***	1.453***	1.192***	1.298***	1.620***	1.659***	1.094***
	(23.58)	(21.07)	(11.13)	(19.19)	(8.89)	(10.45)	(16.55)	(16.24)
Growth	1.908***	2.004***	1.625***	1.925***	2.464***	1.281***	2.557***	1.612***
	(14.76)	(13.20)	(6.59)	(12.48)	(7.46)	(3.75)	(9.52)	(11.12)
Lev	-5.238***	-5.293***	-4.923***	-4.441***	-6.621***	-6.951***	-7.133***	-3.953***
	(-15.39)	(-13.72)	(-6.80)	(-11.03)	(-7.54)	(-7.48)	(-10.66)	(-10.20)
Occupy	-28.246***	-27.608***	-29.554***	-29.939***	-35.214***	-14.452**	-28.362***	-28.698***
	(-12.24)	(-10.30)	(-6.41)	(-10.75)	(-5.93)	(-2.47)	(-6.68)	(-10.55)
Roe	33.199***	33.948***	30.418***	32.411***	35.442***	33.582***	32.871***	33.079***
	(72.23)	(64.70)	(32.10)	(59.63)	(29.71)	(26.88)	(38.10)	(62.08)
_cons	-12.106***	-11.600***	-16.247***	-10.765***	-12.521***	-19.623***	-20.356***	-8.809***
	(-10.79)	(-9.46)	(-5.84)	(-8.29)	(-4.11)	(-5.97)	(-9.40)	(-6.28)
Ind	control	control	control	control	control	control	control	control
Year	control	control	control	control	control	control	control	control
N	14196	11443	2753	10059	2234	1903	4932	9264
R2	0.41	0.42	0.42	0.40	0.45	0.44	0.41	0.42

Note: ***, **, * are significant at the levels of 1%, 5% and 10%, respectively, and the Z value in parentheses.

Column (1) in Table 5 is the full sample test result. The regression coefficient of environmental regulation intensity (ER) and companies non-financial performance (RO) is -0.058, and it is significant at the 1% confidence level, which shows that the government environment regulatory policies inhibit the non-financial performance of enterprises. The H2 hypothesis is verified.

Columns (2) and (3) are the regression results obtained after distinguishing the pollution degree and the pollution natures. The regression coefficient of environmental regulation (ER) and non-financial performance (RO) in heavy pollution industries is -0.063, which is significant at the 1% confidence level. The environmental regulation (ER) and non-financial performance (RO) of light pollution industries show an insignificant negative correlation, with a regression coefficient of -0.029. It can be seen that the government's environmental regulation policies have different effects on enterprises with different pollution levels and pollution natures. The government's environmental regulatory pressure has brought more significant performance suppression effects to heavy pollution industries, while the suppression effect on light pollution industries is not obvious. The H2a hypothesis is verified.

Columns (4), (5), and (6) are the regression test results after subdivide companies locations. The regression coefficients of environmental regulations (ER) and non-financial performance (RO) in eastern, central, and western regions are -0.044, -0.053, -0.079, respectively. Among them, the eastern and western regions are both significant at the 1% confidence level, and the central region is significant at the 5% confidence level. It can be seen that there are regional differences in the effects of the government's environmental regulation policies. Environmental regulations have a significant inhibitory effect on the eastern, central, and western regions, with the strongest inhibitory effect on the western region, followed by the central region, and then the eastern region. The reason is that the level of companies non-financial performance (RO) is greatly affected by regional trade conditions. Due to the more developed economic level, stronger social resources, and more advantageous location conditions in the eastern region, there are more ways to respond when faced with environmental regulatory pressures. These advantages can offset the inhibitory effects of environmental regulations under certain conditions. The H2b hypothesis is verified.

Columns (7) and (8) are the inspection results after distinguishing the nature of property rights. The regression coefficients of the non-financial performance (RO) of state-owned and non-state-owned enterprises to environmental regulation are -0.072 and -0.041, respectively, which are significant at the 1% confidence level. However, the inhibitory effect of environmental regulation on performance in state-owned enterprises is more obvious than that of non-state-owned enterprises. The H2c hypothesis is verified.

From the point of view of control variables, enterprise size (Size), company growth (Growth), return on net assets (Roe) with the company's non-financial performance (RO) show a positive correlation at the 1% confidence level. Leverage ratio (Lev), major shareholder's capital occupation (Occupy) with companies non-financial performance (RO) show a negative correlation at the 1% confidence level. It shows that the larger the scale of the company, the better the growth of the company, and the higher the return on net assets of the company, the easier it is to obtain a good level of non-financial performance. However, it is not easy to achieve good non-financial performance improvement when the company's leverage ratio or major shareholders’ capital are high.

### Robustness analysis

In order to make the research results of this article more rigorous and reliable, this article replaces the measurement method of explanatory variables (Environmental regulation) and does the following robustness test. Use the "average value of pollution control at the location of the company" to replace the aforementioned measurement of "1000* pollution control investment/GDP" for testing.

Table 6 reports the results of the robustness test, ER1 = mean value of pollution control at the location of the company. The test result of Model 1 shows that the correlation coefficient between the intensity of environmental regulation (ER1) and companies financial performance (RFI) is -0.097, which is significant at the 1% confidence level, indicating a significant negative correlation between the intensity of environmental regulation and companies financial performance. The test result of Model 2 shows that the correlation coefficient between the intensity of environmental regulation (ER1) and the non-financial performance (RO) of the company is -0.121, which is significant at the 1% confidence level, indicating that the intensity of environmental regulation and the non-financial performance of the company are significantly negative relationship. This shows that it is basically consistent with the previous conclusions and the model assumptions are robust.

**Table 6:** Robustness test.

	Model 1	Model 2
	RFI	RO
ER1	-0.097***	-0.121***
	(-6.76)	(-9.92)
Size	1.933***	1.203***
	(27.82)	(22.32)
Growth	-0.672***	1.862***
	(-4.46)	(14.41)
Lev	-6.442***	-4.998***
	(-15.01)	(-14.67)
Occupy	20.156***	-27.721***
	(7.24)	(-12.05)
Roe	2.483***	33.333***
	(4.56)	(72.64)
_cons	-27.209***	-10.137***
	(-18.36)	(-8.85)
Ind	Control	Control
Year	Control	Control
N	14196	14196
R2	0.10	0.42

## Conclusion

Using data from 2839 Chinese listed companies from 2014 to 2018, this paper explored the differences in the impact of environmental regulations on companies financial performance and non-financial performance. The research conclusions are as follows:

1. Environmental regulations are negatively related to companies financial performance. The government's environmental regulation policies have a restraining effect on the financial performance of enterprises. There are significant differences in the effects of government environmental regulation policies on enterprises with different pollution levels and pollution natures. The government's environmental regulatory pressure has brought significant performance restraint effects to the financial performance of heavy pollution industries, but has little effect on the financial performance of light pollution industries.
2. Environmental regulations are negatively related to non-financial performance of enterprises. The government's environmental regulation policies have a restraining effect on the non-financial performance of enterprises. There are significant differences in the effects of government environmental regulation policies on enterprises with different pollution levels and pollution properties. The government's environmental regulatory pressure has brought a more significant performance suppression effect to the heavy pollution industries, while the suppression effect on the light pollution industries are not obvious. There are regional differences in the effects of the government's environmental regulatory policies, with the strongest restraining effect in the western region, while its restraining effect is relatively weakened in the eastern region.
3. The impact of environmental regulations on the performance of financial and non-financial differs among enterprises with different property rights. Compared with state-owned enterprises, environmental regulations have a significant inhibitory effect on the financial performance of non-state-owned enterprises; Environmental regulations have a restraining effect on the non-financial performance of both state-owned and non-state-owned enterprises, but the restraining effect of environmental regulations on state-owned enterprises is greater than that of non-state-owned enterprises.

### Policy suggestion

The government should improve environmental laws and regulations to adapt measures to local conditions. The government's environmental regulation policies have different degrees of influence on companies with different geographic regions, different pollution levels, and different property rights. Therefore, when formulating environmental laws and regulations, it is necessary to formulate scientific and reasonable environmental regulatory policies in a targeted manner.

Generally speaking, environmental regulations should not only be a top-down control method, and they cannot be issued only as one-way indicators. What is more important is to explore, pilot and promote various environmental supervision methods, and carry out multi-pronged management methods on environmental issues to maximize the flexibility of environmental supervision policies and explore the following ideal situations: Under the continuous improvement of public environmental awareness and the gradual improvement of the green product market, companies actively carry out pollution control, take proactive measures to meet the challenges of internalization of environmental costs, and achieve a "win-win" situation between environmental protection and companies benefits.
